# Clinical outcomes after reverse shoulder arthroplasty in patients 60 years old and younger; medium-term results

**DOI:** 10.1016/j.jseint.2022.12.018

**Published:** 2023-01-13

**Authors:** Aaron M. Chamberlain, Alexander W. Aleem, Julianne A. Sefko, Karen Steger-May, Jay D. Keener

**Affiliations:** a Department of Orthopaedic Surgery, Washington University Medical Center, Saint Louis, MO, USA

**Keywords:** Reverse, Shoulder arthroplasty, Young patients, Patient reported outcome, Revision shoulder arthroplasty, Radiographic outcomes

## Abstract

**Background:**

Reverse total shoulder arthroplasty (RTSA) has been well-described as a surgical solution to manage rotator cuff tear arthropathy in elderly, low demand paitents. As experience has increased along with improvements in technique and implant design, RTSA has become increasingly used to manage more varied pathologic conditions of the shoulder in younger, more active patients. This study evaluates outcomes in a consecutive series of patients aged 60 years old and younger after undergoing RTSA.

**Methods:**

There were 94 shoulders in 89 patients enrolled. Mean age of the cohort was 54.8 (range 18-60 years). Surgical indications included rotator cuff tear arthropathy, irreparable rotator cuff tear without arthritis, glenohumeral arthritis with erosive glenoid deformity, inflammatory arthropathy, proximal humerus fracture nonunion/malunion and failed prior shoulder arthroplasty. Sixty-one shoulders (70%) had undergone at least one prior surgery. Of these, 6 shoulders (6% of total cohort) had a prior failed arthroplasty. Clinical outcomes (American Shoulder and Elbow Surgeons score, Western Ontario Osteoarthritis of the Shoulder index; visual analog scale pain), radiographic outcomes and complications were analyzed and assessed for correlation with patient demographic factors.

**Results:**

The mean follow-up for this cohort was 4.9 years (range 2-12 years). Subjects experienced improvements in ASES score and pain (*P* < .001) and active forward elevation (88° preop to 135° postop, *P* < .001). Prior operation correlated with worse postoperative ASES and WOOS scores. Higher demand occupation correlated with less improvement in pain scores. The overall complication rate was 12%. Seven shoulders (7%) underwent an additional procedure. There was a 2% incidence of dislocation and a 4% incidence of acromial stress fracture. There was a 36% incidence of notching.

**Conclusion:**

With medium-term follow-up, RTSA is a reliable and predictable operation to manage various pathologic conditions in patients aged 60 years or less. Patients predictably experience significant improvements in pain and range of motion while assuming a modest complication risk. Long-term study is needed to understand potential for late complications or implant failure.

The durability and survivorship of reverse total shoulder arthroplasty (RTSA) in young patients are unknown and represent a potentially significant clinical challenge given the rapidly expanding indications for this surgery. Since the first RTSA implant was cleared by the United States Food and Drug Administration (FDA) in 2003, RTSA has become a well-established surgical technique to manage severe rotator cuff disease in older patients.^9^^,^^19^^,^^20^ Due to concerns for implant loosening, the early indications for RTSA were limited to older or lower demand patients with severe cuff-related disease. Early published results of RTSA in elderly, low demand patients with cuff tear arthropathy noted reliable improvements in pain, range of motion, and overall functional status.^2^^,^^10^

As techniques and implant designs have improved, the use of RTSA has expanded beyond cuff tear arthropathy. Studies have shown that RTSA can be used to successfully manage proximal humerus fractures and fracture sequelae, glenohumeral osteoarthritis, arthroplasty in the setting of glenoid bone deficiency, and revision arthroplasty.^3^^,^^13^^,^^16^^,^^25^^,^^26^ Short-term and mid-term outcomes have generally been favorable. While the use of RTSA has expanded, so too have advanced age and low physical demand become less strict patient selection criteria. A few studies have described outcomes in small groups of younger patients undergoing reverse shoulder arthroplasty with predictable short and mid-term clinical outcomes.^7^^,^^18^^,^^22^^,^^23^ The question of longer term durability in these patients remains unanswered. Ernstbrunner et al published long-term results (mean 11.7 years) of a small series of young patients who underwent RTSA and experienced reliable and sustained clinical outcomes and implant survivorship; however, a relatively high complication rate (39%) was described.

Time-dependent complications as well as concerns over implant survivorship and functional longevity beyond the short and mid-term remain a concern. Guery et al and others recently reported that Constant Scores, radiographic results, and survivorship deteriorated at a follow-up time of 6 to 8 years.^11^ Bacle et al published a study describing outcomes in elderly patients (mean age 82) who were a mean of 12.5 years from surgery.^1^ Implant survivorship for those in the cohort was 93% and Constant Scores remained improved compared to preoperative scores but the authors noted a significant deterioration in Constant Score when comparing medium to long-term clinical scores. While these studies report outcomes primarily in older patients, relatively little is known about the outcomes of this procedure in a younger, more active population.

The number of patients presenting with shoulder arthritis is rapidly growing as is the number of arthroplasty procedures performed annually.^5^ The incidence of all shoulder arthroplasty procedures (including hemiarthroplasty, anatomic total shoulder arthroplasty (TSA), and reverse shoulder arthroplasty) is increasing steadily in the United States.^14^ In some settings, the reverse shoulder arthroplasty has become the most common type of shoulder arthroplasty.^4^ As patient selection broadens and surgical techniques continue to evolve, the number of reverse shoulder arthroplasty procedures performed annually is expected to continue to rise and increasingly be performed in younger patients.

The purpose of this study is to assess the mid-term outcomes following RTSA in patients sixty years or younger at the time of surgery for the purpose of characterizing these patients, their indications for surgery, and evaluating their clinical and radiographic outcomes and complication rates. It was hypothesized that RTSA would convey significant and predictable improvement in clinical outcomes in the population age 60 years and younger in both the primary and revision setting. This early to mid-term follow-up study will serve as a baseline against which future longer-term comparisons of this cohort can be performed.

## Materials and methods

Approval for this single institution retrospective cohort study was obtained from our Institutional Review Board. Hospital operative records and patient charts were reviewed to identify patients who met inclusion criteria. Patients were then contacted, invited and consented to participate in the study. All subjects had undergone RTSA performed by one of four fellowship trained shoulder and elbow surgeons at our tertiary orthopedic center. Inclusion criteria were having undergone a reverse total shoulder arthroplasty for any indication at the age or 60 or younger at least 2 years prior to enrollment. We included both primary and revision arthroplasty surgery. The operative indication for the RTSA was recorded. Revision arthroplasty procedures involved conversion of a failed hemiarthroplasty or anatomic total shoulder arthroplasty to a RTSA. The number of revision procedures, reason for revision, type of revision, and time to revision was also obtained from chart review and document for each patient. Subjects were excluded if they were older than 60 years old at the time of surgery or had less than 2 years of follow-up and if they were unwilling to participate in a final follow-up evaluation. Demographic variables including age, gender, hand dominance, occupation, medical comorbidities (using Charlson Comorbidity Index), history of prior ipsilateral shoulder surgery were all assessed. Involvement of a Worker’s Compensation claim, litigation clam and disability claim were also noted. American Shoulder and Elbow Surgeons (ASES) score and visual analog scale (VAS for pain) were obtained preoperatively. Preoperative range of motion was obtained from documented physical exam in the patient chart.

### Surgical technique

All surgeries were performed through a deltopectoral approach. Of 94 patients, 90 (96%) underwent RTSA with a central post baseplate with Trabecular Metal coating (TM Reverse; Zimmer Biomet, Warsaw, IN, USA) and 4 (4%) with a central post baseplate by Tornier/Wright Medical (Wright Medical, Memphis, TN, USA). The humeral implant was a Zimmer-Biomet TM Reverse shoulder stem in 93 shoulders (99%) and Tornier Aequalis in 1 (1%) (Table I). The humeral components in these designs have a humeral angle of inclination of either 150° or 155°. All Zimmer TM reverse glenoid baseplate provide between 2 and 4 mm of lateralization depending on the baseplate selected. The humeral stem was preferably placed uncemented when the bone quality was adequate. The subscapularis was selectively repaired only if the tissue quality and excursion were deemed satisfactory at the time of surgery. Intraoperative details including implant size details, glenoid defects, if any, and the need for structural glenoid bone graft were recorded from the operative report.

**Table I:** Outcomes summary.

Mean f/u (y)	5.0 y (2-12)		
Implant type	Zimmer TM Reverse	Tornier Aequalis	
Metaglene/Glenosphere	90	4	
Humeral Stem	93	1	
Range of Motion	Preoperative	Postoperative	*P* value
Forward Elevation	87 ± 40.6	135 ± 14.3	*P* < .0001†
External Rotation	32 ± 19.6	33 ± 16.4	*P* = .52
Clinical Outcome Scores			
ASES score	27.3 ± 17.4	74.1 ± 18.9	*P* < .0001†
VAS pain∗	7.1 ± 2.1	1.4 ± 2.1	*P* < .0001†
Notching (Sirveaux Grade)			
0	64%		
1	23%		
2	10%		
3	2%		
4	1%		
Complications	11 (12%)		
Reoperations	7 (7%)		
Acromial Stress Fracture	4 (4%)		

*f/u*, follow up; *ASES*, American Shoulder and Elbow Surgeons; *VAS*, visual analog scale.

∗ Data collected on a visual analog scale where 0 = no pain at all and 100 = pain as bad as it can be. Converted to 0-10 scale.

† *P* values were based on shoulders with data at both pre- and postoperative visits.

**Table II:** Demographic variables.

Demographic variables	N∗	Summary statistic	Range
Age, at time of RSA surgery (y), mean (SD)	94	54.8 (6.5)	18.0 to 60.7
Female gender, no. (%)	94	58 (61.7%)	
Race†, no. (%):	94		
Black or African American		3 (3.2%)	
Native Hawaiian or Other Pacific Islander		1 (1.1%)	
White		89 (94.7%)	
Unknown/Refused		1 (1.1%)	
Ethnicity†, no. (%)	94		
Hispanic or Latino		0 (0%)	
Not Hispanic or Latino		93 (98.9%)	
Unknown/Refused		1 (1.1%)	
Enrolled shoulder is the dominant side‡, no. (%)	94	64 (68.1%)	
Smoking status, no. (%)	91		
Current smoker		15 (16.5%)	
Previous smoker		27 (29.7%)	
Never smoked		49 (53.8%)	
Charlson Comorbidity Index, median (IQR)	94	1.0 (2.0)	0 to 6
Self-identification as retired, no. (%)	92	52 (56.5%)	
Physical demands classification reported at post-op§, no. (%)	92		
Sedentary		55 (59.8%)	
Light work		15 (16.3%)	
Medium work		13 (14.1%)	
Heavy work		5 (5.4%)	
Very heavy work		4 (4.4%)	

*N*, number of shoulders; *RSA*, reverse shoulder arthroplasty; *IQR*, interquartile range; *y*, year; *SD*, standard deviation.

∗ Number of shoulders with non-missing data for the specified variable.

† Self-identified race is captured with several “check all that apply” categories (data not shown). For race and ethnicity, refusals are categorized as “Unknown/Refusal” rather than as missing data.

‡ Data are captured by assessing the enrolled shoulder (right/left) and the dominant hand (right/left/both). When both sides are reported to be dominant, the enrolled shoulder is considered the dominant side. When the dominant hand is missing at preop, the postop value is used.

§ Data collected by guided interview and is collected regardless of employment status using the U.S., Department of Labor, Employment and Training Administration (1991) Dictionary of Occupational Titles. The physical demands strength rating reflects the estimated overall strength requirement of the job. An evaluation is made of involvement in standing, walking, and sitting.

### Clinical examination

Attempts were made to contact all eligible patients by phone and/or mail to invite them to participate in a current clinical and radiographic assessment. Subjects who agreed to participate in the study completed a consent. All subjects were asked to complete the ASES and VAS score for pain in addition to the Western Ontario Osteoarthritis of the Shoulder (WOOS) and SF-12 questionnaires. Charts were also reviewed to asses for any postoperative complications (infection, dislocation, fracture, implant failure, and wound complications) and return to the operating room.

Subjects who returned for physical examination underwent range of motion examination to assess active shoulder motion to include forward elevation, external rotation in adduction, external rotation at 90 degrees of scapular plane elevation and internal rotation behind the back. Subjects who were unable to return for a clinical visit performed a previously validated self-reported range of motion assessment.^29^

### Radiographic examination

Preoperative and postoperative radiographs were reviewed and graded by 2 attending orthopedic surgeons. In cases of rotator cuff tear arthropathy, disease severity was graded using the classification by Hamada.^12^ Preoperative CT scans and MRIs were corrected to the scapular plane assessed when available. Glenoid version was measured at the level of the coracoid tip on axial series referencing Friedman’s line. Postoperative radiographs were examined to determine the presence of glenoid and humeral component loosening or subsidence as well as glenoid notching as defined by Sirveaux.^24^ Humeral radiolucencies were graded using the zone system analogous to that of Gruen et al for THA.^17^ When present, acromial or scapular spine stress fractures were classified using the Levy classification.^15^

### Statistical analysis

Paired t-tests were used to determine if outcomes significantly changed between pre- and postoperative. Spearman correlation coefficients assessed the association of (a) demographics, surgical/clinical characteristics, and complications with postoperative outcomes and change in outcomes, and (b) demographics, surgical/clinical characteristics, and preoperative outcomes with postoperative satisfaction. Postoperative outcome scores were compared for shoulders with and without sustained acromial stress fractures by Wilcoxon’s test. Sample sizes vary across pre- and postoperative variables; analyses used available data from each shoulder. The analysis includes bilateral data from five patients. There was no adjustment for the lack of independence of data from the same patient due to the limited sample size. A *P*-value of < .05 was considered significant. All statistical analyses were performed using SAS software, version 9.4 of the SAS System for Windows (SAS Institute Inc., Cary, NC, USA).

## Results

From January 2006 to May 2017 we identified 152 consecutive patients who underwent a reverse shoulder arthroplasty at the age of 60 years old or younger at our institution. Of these patients, 7 were deceased at the time of attempted contact. An additional 18 declined participation citing ongoing illness or injury and 16 declined for unspecified reasons. Of the remaining 111 potential subjects, 22 were unable to be successfully contacted after multiple attempts. All remaining 89 subjects were enrolled in the study. Five subjects enrolled both right and left shoulders in the study; thus, 94 shoulders were included and analyzed.

Demographic analysis of our cohort found that 62% (n = 58) were female. Subjects had an average age of 54.8 years at time of surgery (range, 18.0-60.7 years). Mean follow-up for the cohort was 4.9 years (range, 2-12 years). Median follow-up was 4.3 years (interquartile range = 3.6). Of the 70 potentially eligible shoulders not enrolled, the average age at the time of surgery was 54.3 years (range 27-59 years) with a mean follow-up time of 27.5 months (range 1-121 months). Sixty-four enrolled shoulders (68%) had undergone reverse shoulder replacement on the dominant side. Fifteen shoulders (16%) were in current smokers, 27 (30%) in previous smokers and the remaining (54%) had never smoked. Nine shoulders (10%) were in subjects who classified their physical demands at work as either ‘Heavy’ or ‘Very Heavy’. Fifty-five shoulders (60%) were in subjects who reported mainly sedentary physical demands (Table II).

### Indications

**Figure 1:**
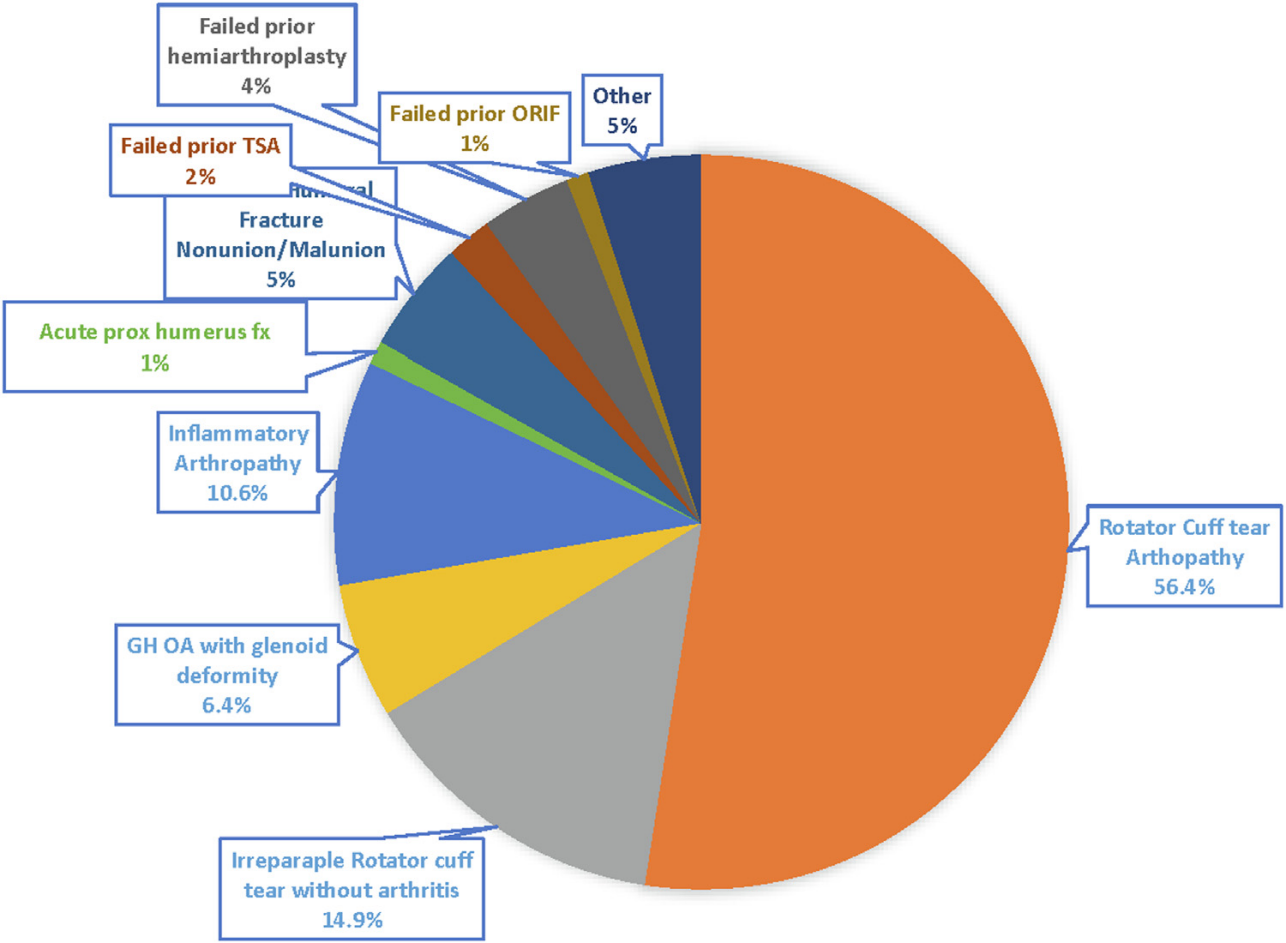
Indications for reverse shoulder arthroplasty. *ORIF*, open reduction internal fixation; *TSA*, total shoulder arthroplasty; *GH OA*, glenohumeral osteoarthritis.

Indications for reverse shoulder arthroplasty included rotator cuff tear arthropathy (56%), irreparable rotator cuff tear without arthritis (15%), glenohumeral osteoarthritis with glenoid erosive deformity (6%), inflammatory arthropathy (11%), proximal humeral fracture sequelae including nonunion/malunion (5%). Four subjects had a failed prior hemiarthroplasty, 2 had failed prior anatomic total shoulder arthroplasty and 1 had failed prior fracture fixation. One subject underwent RTSA for an acute proximal humerus fracture (Fig. 1). Sixty-one subjects (70%) had a prior operation on the enrolled shoulder. Thirty-three (35%) had one prior operation, 20 (21%) had 2 prior operations, 7 (7%) had 3 prior operations and 1 (1%) had 4 prior operations (Table III). The subscapularis was repaired in 8 shoulders. Ten subjects had glenoid deficiencies that were managed intraoperatively with a structural bone graft. Of these 10 cases, 3 used humeral head autograft and 7 used femoral head allograft. Of these ten subjects, 5 were performed as a revision arthroplasty procedure (3 were revisions of failed hemiarthroplasties and 2 were failed TSAs).

**Table III:** Prior operations.

Prior operations	n
1 Prior operation	
Rotator Cuff repair	23
Instability surgery	2
Arthroscopic débridement	2
ORIF fracture	3
Tissue/Bone Biopsy	2
Hemiarthrosplsty	1
Total	**33**
2 Prior Operations	
Rotator cuff repair × 2	8
Rotator cuff repair; Arthroscopic débridement	2
Arthroscopic stabilization, arthroscopic fx fixation	1
Arthroscopic débridement; latissimus dorsi transfer	1
Rotator cuff repair; Hemiarthroplasty	1
Rotator cuff repair; latissimus dorsi transfer	2
Arthroscopic débridement; Hemiarthroplasty	1
Rotator cuff repair; Deltoid-plasty	1
Hemiarthroplasty; I&D with antibiotic spacer placement	1
Anterior capsule reconstruction; pectoralis major transfer	1
ORIF proximal humerus fracture; Hemiarthroplasty	1
Total	**20**
3 Prior Operations	
Rotator cuff repair × 3	5
Hemiarthroplasty; revision to TSA; pec major transfer	1
Rotator cuff repair × 2; I&D for infection	1
Total	**7**
4 Prior Operations	
Rotator cuff repair × 4	1

*ORIF*, open reduction internal fixation; *TSA*, total shoulder arthroplasty; *I&D*, irrigation and débridement.

### Complications

A total of 7 subjects (7%) underwent an additional surgery at a mean of 20.5 months after index reverse shoulder arthroplasty (Table IV). Of the surgical revisions, there were two cases (2%) of prosthetic instability requiring revision and two subjects developed hematomas. Two subjects (2%) had aseptic loosening of the metaglene. One additional subject underwent repeat surgery over 6 years from the index procedure to remove symptomatic broken wires that were originally used to fix the fractured greater tuberosity. When including acromial stress fractures (4 subjects) as a complication, the overall complication rate in this cohort was 12% (Table I). Each of the four acromial stress fractures were classified as Levy type I.

**Table IV:** Complications.

Complications	Indication for index RTSA	Time to reoperation (mo)	Comments
Instability (<6 weeks)	Irreparable Rotator Cuff tear without arthritis	1	Treated definitively with polyethylene liner exchange
Instability (>6 weeks)	Proximal Humerus fracture malunion/nonunion	5	Treated definitively with polyethylene liner exchange
Nerve parasthesia (Carpal Tunnel)	Rotator Cuff Tear Arthropathy	N/A	Did not require additional surgery
Postoperative Hematoma	Irreparable Rotator Cuff tear without arthritis	<1	Surgical Evacuation of Hematoma
Postoperative Hematoma	Rotator Cuff Tear Arthropathy	<1	Surgical Evacuation of Hematoma
Aseptic loosening of Metaglene	Glenohumeral Osteoarthritis	17	Index procedure with structural allograft bone
Aseptic loosening of Metaglene	Failed Anatomic Total Shoulder Arthroplasty (Revision)	41	Index procedure with structural allograft bone
Painful/broken wire	Proximal Humerus fracture malunion/nonunion	78	Removal of painful/broken wire
Acromial Stress Fracture	Rotator Cuff Tear Arthropathy	N/A	Managed nonoperatively
Acromial Stress Fracture	Irreparable Rotator Cuff tear without arthritis	N/A	Managed nonoperatively
Acromial Stress Fracture	Rotator Cuff Tear Arthropathy	N/A	Managed nonoperatively
Acromial Stress Fracture	Rotator Cuff Tear Arthropathy	N/A	Managed nonoperatively

*RTSA*, reverse total shoulder arthroplasty.

### Clinical outcomes

Preoperatively, 66 completed the ASES questionnaire and 69 completed the VAS pain score. The mean preoperative ASES score was 27.3 (SD 17.4) and the mean preoperative VAS pain score was 7.1 (SD 2.1). Ninety subjects completed the ASES, VAS pain and WOOS postoperatively. The mean ASES score postoperatively was 74.1 (SD 18.9), the mean postoperative VAS pain score was 1.4 (SD 2.1) and the mean postoperative WOOS score was 70.9 (SD 23.5). For those patients who completed ASES and VAS pain surveys both preoperatively and postoperatively the improvements after surgery were both statistically significant (*P* < .0001). Subjects were also asked at final follow-up about their satisfaction with the procedure. Ninety five percent of subjects were satisfied with the outcome of the procedure and 91% of subjects responded that they would undergo the same treatment for their shoulder problem if they could go back in time.

Preoperative active forward elevation motion data was available for 87 subjects and external rotation motion (at the side) data was available for 75 subjects. Mean active forward elevation was 88° (SD 40.6°). Mean External rotation at the side was 32° (SD 19.6°). Internal rotation motion was not consistently reported in the preoperative clinical examination. Final follow-up postoperative range of motion was measured clinically in 73 subjects. Mean forward elevation in this group was 135° (SD 14.3°) and external rotation at the side was 33° (SD 16.2°). The postoperative internal rotation behind the back values were: to T5 in 4 subjects (6%), T7 in 12 (16%), thoracolumbar junction in 28 (38%), belt line in 10 (14%), buttock in 12 (16%) and the ipsilateral side in 7 (10%). Improvements in active forward elevation were statistically significant (*P* < .0001) while improvements in external rotation at the side were not significant (*P* = .52). An additional 21 subjects were not able to return for clinical evaluation in person and instead completed the range of motion self-assessment. Of these, 14 (70%) rated their active forward elevation of the shoulder as full or nearly full (>135°) and 16 (80%) were able to externally rotate at the side to between 20° and 40°.

### Radiographic outcomes

Of the 53 patients with the operative indication of rotator cuff tear arthropathy, 18 (34%) were classified as Hamada Grade 1, 2 (4%) were Grade 2, 6 (11%) were Grade 3, 4 (8%) were Grade 4 and 23 (43%) were Grade 5. Preoperative CT scan was available in 27 patients and MRI was available in 45 patients. Mean preoperative glenoid version was 5.8° (SD 8.4) of retroversion. Postoperative radiographs were available in 83 subjects at a mean of 4.2 years postoperatively. Two subjects experienced subsidence of the baseplate and required revision. Both had a reverse shoulder arthroplasty performed with structural bone graft to address glenoid bone deficiency. Two other subjects had radiographic evidence of lucency around the central peg of the baseplate with no sign of subsidence. One of these also demonstrated lucency behind the baseplate.

Notching was assessed at final follow-up. Fifty-three (64%) had no evidence of notching; 19 (23%) had grade 1; 8 (10%) had grade 2, 2 (2%) had grade 3 and 1 shoulder (1%) had grade 4 (Table I). There was no statistically significant correlation between no or minimal notching (grade 0 or 1) vs. higher grade (grades 2, 3 and 4) notching and postoperative ASES, WOOS or VAS scores. There was a correlation between higher grade notching and more active external rotation at the side (*ρ* = +0.29, *P* = .01) (Table V).

**Table V:** Correlation with postoperative outcomes (Spearman correlation coefficient).

Preoperative factors	Postop VAS pain	*P* value	Postop ASES Score	*P* value	Postop WOOS Score	*P* value	Postop Active Forward Elevation-active	*P* value	Postop Active External Rotation at side	*P* value

Gender 1 = male, 2 = female	0.02874	.7868	−0.03842	.7192	0.03508	.7413	−0.15801	.1818	−0.00663	.9556
Occupation (Physical Demand) 1 = sedentary, 5 = very heavy	−0.01406	.8947	0.14313	.1784	0.11507	.2774	0.19382	.1004	0.17819	.1315
Preop Hamada grade (1 = grade 1, 5 = grade 5)	0.10878	.3075	−0.07257	.4992	−0.13717	.1973	−0.18538	.1164	0.00629	.9579
Preop Glenoid retroversion	0.14904	.2182	−0.03809	.756	−0.01253	.918	−0.11106	.4195	0.01753	.8989
Structural Bone graft used (1 = No, 2 = Yes)	0.04426	.677	−0.00642	.9521	−0.03879	.7151	−0.01987	.8675	−0.01488	.9005
Surgical Indication (1 = No, 2 = Yes)										
Rotator Cuff Tear Arthropathy	0.06534	.5383	−0.10631	.3186	0.02943	.7818	−0.10138	.3934	−0.0927	.4354
Irreparable Rotator Cuff tear without Arthropathy	0.15652	.1384	−0.11369	.286	−0.18589	.0777	0.05448	.6471	−0.13168	.2668
Glenohumeral Osteoarthritis	−0.14688	.1647	0.12045	.2581	0.25281	**.0156**	0.34376	**.0029**	0.13593	.2515
Inflammatory Arthropathy	−0.10664	.3144	0.11092	.298	0.15048	.1545	−0.0532	.6549	0.12903	.2766
Failed Prior Surgery	0.01338	.8998	0.07116	.5051	−0.06437	.5444	−0.05302	.656	0.01808	.8793
Failed Prior Arthroplasty	−0.06745	.5252	0.0263	.8056	−0.07848	.4597	−0.05302	.656	0.01808	.8793
Prior Operation (1 = No, 2 = Yes)	0.19153	.069	−0.21669	**.0402**	−0.28299	**.0066**	0.07104	.5504	−0.12929	.2757
Increasing Number of Prior Operations	0.17777	.0918	−0.15141	.1543	−0.24452	**.0195**	0.03931	.7413	−0.10642	.3702
Postop Notching: None/minimal (Grades 0,1 = 0) vs. Higher grade (Grades 2,3,4 = 1)	0.0182	.8711	−0.01285	.9088	0.02721	.8083	−0.12903	.2835	0.29047	**.014**

*Postop*, post operative; *VAS*, visual analog scale; *ASES*, American Shoulder and Elbow Surgeons; *WOOS*, Western Ontario Osteoarthritis of the Shoulder index.

Statistically Significant values (*P* < .05) in Bold.

Fifty-eight (70%) of stems were press-fit (uncemented) and the remaining 25 (30%) were cemented. Thirty-one (38%) of all stems demonstrated lucency in at least one zone at final radiographic follow-up. Twenty-seven of the stems with lucency (87%) had lucency in only zone 4 (near the distal tip of the implant) and the lucency measured 1 mm or less in all instances. Four other cases demonstrated lucencies in 2 zones all of which had lucency of 1 mm or less in all cases. There were no cases of humeral stem subsidence or gross loosening in this cohort.

### Outcome correlations

Clinical outcomes were examined in relation to various subject factors and preoperative function. No significant correlation was identified in change or improvement (pre/post) of ASES score, pain or ROM when correlated with preoperative age, gender, operative indication, prior surgical procedure or prior arthroplasty surgery. Subjects with a history of prior surgery had significantly worse overall postoperative ASES (*ρ* = −0.22, *P* = .04) and WOOS (*ρ* = −0.28, *P* = .007) scores. Increasing number of prior operations correlated with worse overall postoperative WOOS scores (*ρ* = −0.24, *P* = .019) There was a positive correlation (*ρ* = 0.25, *P* = .04) indicating that more physically demanding occupations were associated with less improvement in pain as measured by the VAS pain score from pre- to postoperative. The surgical indication of glenohumeral osteoarthritis was positively correlated with improved overall postoperative WOOS scores (*ρ* = 0.25, *P* = .016) and active forward elevation (*ρ* = 0.34, *P* = .003) (Table V). Clinical outcomes scores for the 4 subjects who sustained acromial stress fractures did not differ to a significant degree compared to those who did not sustain this complication.

## Discussion

As reverse shoulder arthroplasty becomes increasingly utilized to manage an expanding number of surgical indications in increasingly active patients, close clinical observation is critical to understanding patient safety and clinical outcomes. In the relatively short period of time that the reverse shoulder arthroplasty has been available in the United States, most of the published outcomes studies in the literature have focused on elderly patients being treated for rotator cuff tear arthropathy.^10^^,^^11^^,^^20^^,^^24^^,^^27^^,^^28^ Existing literature regarding younger, more active patients undergoing reverse shoulder arthroplasty is relatively small.^6^^,^^7^^,^^16^^,^^19^^,^^20^ Concerns regarding complication rates and implant survivorship have been cited as limiting factors precluding young, active patients from undergoing RTSA. A few small studies in patients younger than 70 years have described short and mid-term outcomes after RTSA in this younger patient population^7^^,^^18^^,^^21^, ^22^, ^23^ (Table VI). The present study examines the patient demographics, indications and short to mid-term outcomes after RTSA in patients aged 60 or younger. Noteworthy findings of the current study are predictable improvements in pain, shoulder function and high rates of patients satisfaction at mid-term follow-up. Prior surgery of the affected shoulder appears to affect functional outcomes and clinically relevant complications were seen in 12% of patients. To our knowledge, this study is the largest cohort study evaluating medium-term outcomes in patients undergoing RTSA in this age range.

**Table VI:** Previously published studies of outcomes after RSA in young patients.

Study	Ernstbrunner/Gerber 2017		SershonNicholson 2014		Muh/Gobezie 2013		Samuelsen/Sperling 2017	

Sample size	23		36		67		67	
Mean age	57 y (47-59)		54.4 y (V-38 y)		52.2 y (23-60)		60 (50-65)	
Mean f/u	11.7 y (8-19 y)		2.8 y (2-4 y)		3 y (2 y-6.4 y)		3 y (2-8 y)	
Implant type	Grammont (Delta [14], Zimmer Anatomical [9])		Not reported		Grammont (Tornier)		Biomet Comprehensive (40), Delta Xtend (16) and Delta III (3), Aequalis (6), Encore (2)	
	Preoperative	Postoperative	Preoperative	Postoperative	Preoperative	Postoperative	Preoperative	Postoperative
ROM								
Forward elevation	64 ± 32	117 ± 34	57 ± 28	121 ± 46	54.6 (0-165)	134 (0-180)	57.5	132.4
External rotation	28 ± 26	26 ± 19	23 ± 19	30 ± 17	10 (−20 to 70)	19.6 (−10 to 70)	20.1	39.4
Clinical Outcome Scores								
ASES			31.4 ± 18.4	65.8 ± 20.6	40.0 ± 16.71	72.4 ± 12.75	not reported	62 ± 16
VAS (pain)			6.0 ± 3.1	2.1 ± 2.0	7.5 ± 2.0	3.0 ± 2.3		
Constant Score	24 ± 9	59 ± 19						
SSV	20 ± 13	71 ± 27						
SANE			24.4 ± 14.3	72.0 ± 20.9				
SST			1.4 ± 16	6.2 ± 3.7			not reported	5.9 ± 3
Notching (Hamada grade)								
0	5%		82%		57%		82%	
1	48%		18%		33%		9%	
2	19%		0%		7.5%%		3%	
3	19%		0%		3%%		3%	
4	10%		0%		0%%		3%	
Complication	9 (39%)		6 (18%)		10 (15%)		6 (9%)	
Instability	4 (17%)		4 (12%)		5 (7%)			
Reoperation	6 (26%)		4 (12%)		7 (10%)		2 (3%)	

*RSA*, reverse shoulder arthroplasty; *f/u*, follow up; *ROM*, range of motion; *ASES*, American Shoulder and Elbow Surgeons; *VAS*, visual analog scale; *SSV*, subjective shoulder score; *SANE*, single assessment numeric evaluation; *SST*, simple shoulder test.

Smaller short- and medium-term studies in young patients have demonstrated predictably positive clinical outcomes but variably high complication rates. Ek et al published results of a retrospective series of 41 patients under the age of 65 who underwent RTSA to manage rotator cuff tear arthropathy (6). With a mean follow-up of 7.8 years, authors report implant survivorship of 98% and a complication rate of 37.5%.^6^ Muh et al published a similar retrospective study in patients 60 years and younger undergoing RTSA (both primary and revision).^16^ Sixty-seven shoulders were included at a mean follow-up of 2.8 years for primary shoulders and 3 years for revisions. Complication rates were 13.9% for primary RTSAs while revision RTSA had a complication rate of 15%. Samuelson et al published a series of 67 primary RTSAs in patients under the age of 65.^22^ Mean follow-up was 3 years with a complication rate of 9.0%. Implant survivorship was 91% at 5 years.

While early to mid-term improvements in clinical outcomes scores are encouraging, other studies describing longer-term outcomes after RTSA have raised concern about complication rates and diminishing clinical outcomes over time. While some functional deterioration over time may be age-related, concerns of diminishing outcomes over time are especially important to consider in the younger, more active population. Multiple authors have described clinical outcomes that deteriorate in older patients with longer-term follow-up. Guery et al identified worsening functional results and survival after 6 years of follow-up. This study included elderly patients who may have also been experiencing age-related functional decline. Bacle et al reported long-term results after RTSA in patients who underwent surgery to treat a variety of etiologies and included elderly patients (mean age at time of surgery was 72.7 years and mean follow-up was 150 months).^1^ Mean Constant Score was noted to decrease significantly at 10 years postop compared to the scores at 2 years postop. Seventy-three percent of patients demonstrated evidence of scapular notching in this study that included only Grammont style prostheses. The complication rate was 29% and 12% of the cohort underwent reoperation during the follow-up time period. The overall implant survivorship in this series was 93%. Ek et al found that between 5 and 10 years after surgery, implant survivorship decreased in their patients under the age of 60 from 98% to 88%.^6^ Zumstein et al published a meta-analysis in 2011 and identified a comprehensive complication rate of 24% in all age groups.^30^ This high complication rate may be influenced by the fact that this analysis included patients that underwent RTSA for fracture (n = 7.5%) and revision RTSA (27.6%).

Ernstbrunner et al describes the long-term outcomes of a small cohort of 23 shoulders that underwent RTSA under the age of 60 for irreparable rotator cuff tears.^7^ This is the only known outcomes study after RTSA in patients under 60 with long-term follow-up (mean follow-up was 11.7 years). In this study, authors describe sustained clinical improvement over time but with a substantial complication rate. Mean Constant Score and Subjective Shoulder Value had all increased and retained improvement compared to preoperative values as did active anterior elevation and abduction. Importantly, clinical outcomes did not significantly deteriorate after the 10-year time point. The authors did note an increase in notching over time and they found that relative Constant Score was lower in patients where notching was grade 2 or higher. Two (9%) of the RTSAs in this cohort failed during the study period and 39% had at least one complication noted and 26% required reoperation.

In the present study, 36% of patients had evidence of radiographic notching. However, 64% of these were graded as type 1 notching and only 13.5% had evidence of higher grade (types 2, 3 or 4) notching. There were no cases of baseplate subsidence related to notching. While notching is highly dependent upon technique and implant design factors, this rate of notching is similar to the wide range of notching rates that have been published in other studies.^8^^,^^18^^,^^23^^,^^24^ We did not identify any significant difference in patient-reported outcome scores related to higher grades of notching but we did identify diminished external rotation motion at the side. As some studies have identified a possible correlation between notching and worse clinical outcomes, notching remains a concern after RTSA; however, the extent of its clinical significance remains unclear.

The reoperation rate in this cohort was 7% and the overall complication rate was 12%. There were two baseplate failures that were used in the setting of a structural bone allograft to reconstruct the glenoid which required revision. These rates of instability, reoperation and complication are lower than those in most existing studies in the literature. This is notable given that most other studies include only patients with more narrow operative indications (irreparable rotator cuff tear or rotator cuff tear arthropathy). Our study includes patients of varied indications in both the primary and revision setting. That said, it should be emphasized that prior surgery on the shoulder undergoing RTSA was associated with lower postoperative ASES and WOOS scores as was an increasing number of prior surgeries. Also, patients undergoing RTSA for the indication of glenohumeral osteoarthritis in the setting of glenoid bone loss or deformity was moderately correlated with better postoperative WOOS scores compared to those undergoing RTSA for other indications. The comparatively low rates of adverse outcomes and complications in this study are especially encouraging when considering reverse total shoulder arthroplasty in patients under 60 years old who present with varied surgical indications.

This study has limitations that need to be considered. While this is the largest study cohort of this type that we are aware of, the study design is retrospective and is from a single tertiary referral center, potentially limiting generalizability of results to other centers. Furthermore, as a retrospective study we did not prospectively enroll patients prior to surgery. Thus, some preoperative ASES and VAS pain scores are incomplete or missing. This limits our ability to comment on change in preoperative to postoperative ASES scores for approximately 30% of our cohort and VAS pain scores for approximately 27%. Sixty-three of the potential 152 patients who underwent RTSAs at age 60 or less who underwent were not enrolled due to declining the invitation to participate for reasons including declining health, death or the inability to be contacted. While this is a significant number of the potential cohort, the patients who did not enroll were similar to those who enrolled in the study in terms of age and surgical indication, likely minimizing any significant influence they may have had on the results of the study. We do not make any adjustment for the many significance tests performed. As the number of statistical tests increases, the likelihood of achieving a statistically significant result simply by chance also increases. We encourage readers to consider the weight of evidence across variables rather than characterize outcomes on the results of a single variable.

Despite these limitations our study adds to the understanding of outcomes after RTSA in young patients. RTSA provides reliable and predictable improvement in patients under the age of 60 requiring surgical management for a variety of indications. To our knowledge, this is the largest report of a cohort of patients under the age of 60 after RTSA with medium-term follow-up. The study design is also strengthened by the fact that these cases were al performed at a single center by one of 4 fellowship trained Shoulder and Elbow surgeons. This limits potential confounding factors that could be related to differences in surgical technique or surgical experience. In addition, nearly all RTSAs (96%) were performed with the same implant thus reducing the implant-related variability.

## Conclusion

Reverse shoulder arthroplasty is a reliable, predictable option in the short to mid-term for patients aged 60 years old and younger for surgical management of a variety of surgical indications in both the primary and revision setting. Clinical outcomes (ASES and VAS pain) improve significantly after surgery and postoperative WOOS scores achieve satisfactory levels. However, reoperation and revision arthroplasty needs to be considered (7% in this study) although the risk is low. Outcomes may be adversely affected by having a history of prior surgery, and increasing number of prior surgeries. Understanding of long-term maintenance of clinical outcomes (>10 years) remains limited and is of some concern especially in this younger patient population.

## Disclaimers

Funding: This work was supported by 10.13039/100012630Zimmer Biomet (Warsaw, IN, USA). The sponsor reviewed and approved the initial study protocol and final manuscript. They were not involved in the collection, analysis, or interpretation of the data.

Conflicts of interest: Jay Keener reports personal fees from Shoulder Innovations, personal fees from Wright Medical, royalties from Shoulder Innovations and Wright Medical, grants from National Institute of Health, outside the submitted work. Aaron Chamberlain reports personal fees from 10.13039/100007307Arthrex, personal fees from DePuy Synthes, royalties from DePuy Synthes, research grants from 10.13039/100012630Zimmer Biomet, grants from National Institute of Health, outside the submitted work. The other authors, their immediate families, and any research foundation with which they are affiliated have not received any financial payments or other benefits from any commercial entity related to the subject of this article.
